# Transcriptome-wide Mendelian randomization exploring dynamic CD4+ T cell gene expression in colorectal cancer development

**DOI:** 10.1093/jleuko/qiaf131

**Published:** 2025-09-17

**Authors:** Benedita Deslandes, Xueyan Wu, Matthew A Lee, Lucy J Goudswaard, Gareth W Jones, Andrea Gsur, Annika Lindblom, Shuji Ogino, Veronika Vymetalkova, Alicja Wolk, Anna H Wu, Jeroen R Huyghe, Ulrike Peters, Amanda I Phipps, Claire E Thomas, Rish K Pai, Robert C Grant, Daniel D Buchanan, James Yarmolinsky, Marc J Gunter, Jie Zheng, Emma Hazelwood, Emma E Vincent

**Affiliations:** MRC Integrative Epidemiology Unit, University of Bristol, Oakfield House, Oakfield Grove, Bristol BS8 2BN, United Kingdom; Population Health Sciences, Bristol Medical School, University of Bristol, First Floor, 5 Tyndall Avenue, Bristol BS8 1UD, United Kingdom; Department of Endocrine and Metabolic Diseases, Shanghai Institute of Endocrine and Metabolic Diseases, Ruijin Hospital, Shanghai Jiaotong University School of Medicine, 227 South Chongqing Road, Shanghai, China; Shanghai National Clinical Research Center for Metabolic Diseases, Key Laboratory for Endocrine and Metabolic Diseases of the National Health Commission, Shanghai National Center for Translational Medicine, Ruijin Hospital, Shanghai Jiaotong University School of Medicine, 227 South Chongqing Road, Shanghai, China; Nutrition and Metabolism Branch, International Agency for Research on Cancer, WHO, 150 cours Alber Thomas, Lyon 69372 CEDEX 08, France; MRC Integrative Epidemiology Unit, University of Bristol, Oakfield House, Oakfield Grove, Bristol BS8 2BN, United Kingdom; Population Health Sciences, Bristol Medical School, University of Bristol, First Floor, 5 Tyndall Avenue, Bristol BS8 1UD, United Kingdom; School of Cellular and Molecular Medicine, University of Bristol, Biomedical Sciences Building, University Walk, Bristol BS8 1TD, United Kingdom; Center for Cancer Research, Medical University of Vienna, Borschkegasse 8a, Vienna 1090, Austria; Department of Clinical Genetics, Karolinska University Hospital, Solna, Stockholm 171 64, Sweden; Department of Molecular Medicine and Surgery, Karolinska Institutet, Karolinska University Hospital, Solna, Stockholm SE-171 76, Sweden; Program in MPE Molecular Pathological Epidemiology, Department of Pathology, Brigham and Women's Hospital, Harvard Medical School, 221 Longwood Ave, Boston, MA 02115, United States; Department of Oncologic Pathology, Dana-Farber Cancer Institute, 450 Brookline Avenue, Boston, MA 02215, United States; Department of Epidemiology, Harvard T.H. Chan School of Public Health, 677 Huntington Avenue, Boston, MA 02115, United States; Department of Molecular Biology of Cancer, Institute of Experimental Medicine of the Czech Academy of Sciences, Videnska 1083, Prague 142 20, Czech Republic; Institute of Environmental Medicine, Karolinska Institutet, Nobels väg 13, Solna, Stockholm, Sweden; Department of Population and Public Health Sciences, University of Southern California, 1845 N Soto St, Los Angeles, CA 90032, United States; Public Health Sciences Division, Fred Hutchinson Cancer Center, 1100 Fairview Ave., Seattle, WA 98109-1024, United States; Public Health Sciences Division, Fred Hutchinson Cancer Center, 1100 Fairview Ave., Seattle, WA 98109-1024, United States; Department of Epidemiology, University of Washington, 1959 NE Pacific St #F262, Seattle, WA 98195, United States; Public Health Sciences Division, Fred Hutchinson Cancer Center, 1100 Fairview Ave., Seattle, WA 98109-1024, United States; Department of Epidemiology, University of Washington, 1959 NE Pacific St #F262, Seattle, WA 98195, United States; Public Health Sciences Division, Fred Hutchinson Cancer Center, 1100 Fairview Ave., Seattle, WA 98109-1024, United States; Department of Laboratory Medicine and Pathology, Mayo Clinic Arizona, Pathology Research Core, Mayo Clinic, 13400 E. Shea Blvd., Scottsdale, AZ 85259, United States; Division of Medical Oncology and Hematology, Princess Margaret Cancer Centre, 610 University Ave, Toronto, Canada ON M5G 2M9; Department of Clinical Pathology, Colorectal Oncogenomics Group, The University of Melbourne, VCCC Building, Level 10/305 Grattan St, Parkville, VIC 3010, Australia; University of Melbourne Centre for Cancer Research, Victorian Comprehensive Cancer Centre, Flemington Road, Parkville, VIC 3010, Australia; Genomic Medicine and Family Cancer Clinic, Royal Melbourne Hospital, 300 Grattan St, Parkville, Melbourne, VIC 3000, Australia; Cancer Epidemiology and Prevention Research Unit, School of Public Health, Imperial College London, South Kensington Campus, London SW7 2AZ, United Kingdom; Nutrition and Metabolism Branch, International Agency for Research on Cancer, WHO, 150 cours Alber Thomas, Lyon 69372 CEDEX 08, France; Department of Epidemiology and Biostatistics, School of Public Health, Imperial College London, White City Campus, 90 Wood Lane, London W12 0BZ, United Kingdom; Department of Endocrine and Metabolic Diseases, Shanghai Institute of Endocrine and Metabolic Diseases, Ruijin Hospital, Shanghai Jiaotong University School of Medicine, 227 South Chongqing Road, Shanghai, China; Shanghai National Clinical Research Center for Metabolic Diseases, Key Laboratory for Endocrine and Metabolic Diseases of the National Health Commission, Shanghai National Center for Translational Medicine, Ruijin Hospital, Shanghai Jiaotong University School of Medicine, 227 South Chongqing Road, Shanghai, China; MRC Integrative Epidemiology Unit, University of Bristol, Oakfield House, Oakfield Grove, Bristol BS8 2BN, United Kingdom; Population Health Sciences, Bristol Medical School, University of Bristol, First Floor, 5 Tyndall Avenue, Bristol BS8 1UD, United Kingdom; MRC Integrative Epidemiology Unit, University of Bristol, Oakfield House, Oakfield Grove, Bristol BS8 2BN, United Kingdom; Translational Health Sciences, Bristol Medical School, University of Bristol, Dorothy Hodgkin Building, Whitson Street, Bristol,BS1 3NY, United Kingdom

**Keywords:** CD4+ T cells, colorectal cancer, gene expression, genetic epidemiology, Mendelian randomization

## Abstract

Recent research suggests higher circulating lymphocyte counts may protect against colorectal cancer (CRC). However, the role of specific lymphocyte subtypes and activation states remain unclear. CD4+ T cells—a highly dynamic lymphocyte subtype—undergo gene expression changes upon activation that are critical to their effector function. Previous studies using bulk tissue have limited our understanding of their role in CRC risk to static associations. We applied Mendelian randomization (MR) and genetic colocalisation to evaluate causal relationships of gene expression on CRC risk across multiple CD4+ T cell subtypes and activation states. Genetic proxies were obtained from single-cell transcriptomic data, allowing us to investigate the causal effect of expression of 1,805 genes across CD4+ T cell activation states on CRC risk (78,473 cases; 107,143 controls). Analyses were stratified by CRC anatomical subsites and sex, with sensitivity analyses assessing whether the observed effect estimates were likely to be CD4+ T cell-specific. We identified 6 genes—*FADS2, FHL3, HLA-DRB1, HLA-DRB5, RPL28,* and *TMEM258*—with strong evidence for a causal role in CRC development (FDR-*P* < 0.05; colocalisation H4 > 0.8). Causal estimates varied by CD4+ T cell subtype, activation state, CRC subsite and sex. However, many of genetic proxies used to instrument gene expression in CD4+ T cells also act as eQTLs in other tissues, highlighting the challenges of using genetic proxies to instrument tissue-specific expression changes. We demonstrate the importance of capturing the dynamic nature of CD4+ T cells in understanding CRC risk, and prioritize genes for further investigation in cancer prevention.

## Teaser

Integrating Mendelian randomization and colocalisation, we identify CD4⁺ T cell subtype- and activation-specific gene expression changes that causally influence colorectal cancer risk, revealing dynamic relationships and potential preventive targets.

## 1. Introduction

Colorectal cancer (CRC) is the third most common cancer and the second leading cause of cancer-related deaths globally.^1^ While lifestyle risk factors such as obesity, alcohol consumption, and smoking are well-established, the underlying biological pathways driving CRC development remain poorly understood.^2^ This gap in knowledge has limited the development of effective preventative or therapeutic interventions for CRC. Emerging evidence suggests that circulating white blood cell (WBC) counts are linked to CRC risk, progression, severity, and mortality.^3–5^ In particular, we have previously shown a potential protective effect of higher numbers of circulating lymphocytes—a type of WBC—on CRC risk.^6^ However, lymphocytes are a heterogeneous group of immune cells with distinct functional roles, and it remains unclear which specific subtypes mediate this protective effect. This highlights the need for further research into the underlying mechanisms.

**Fig. 1.**
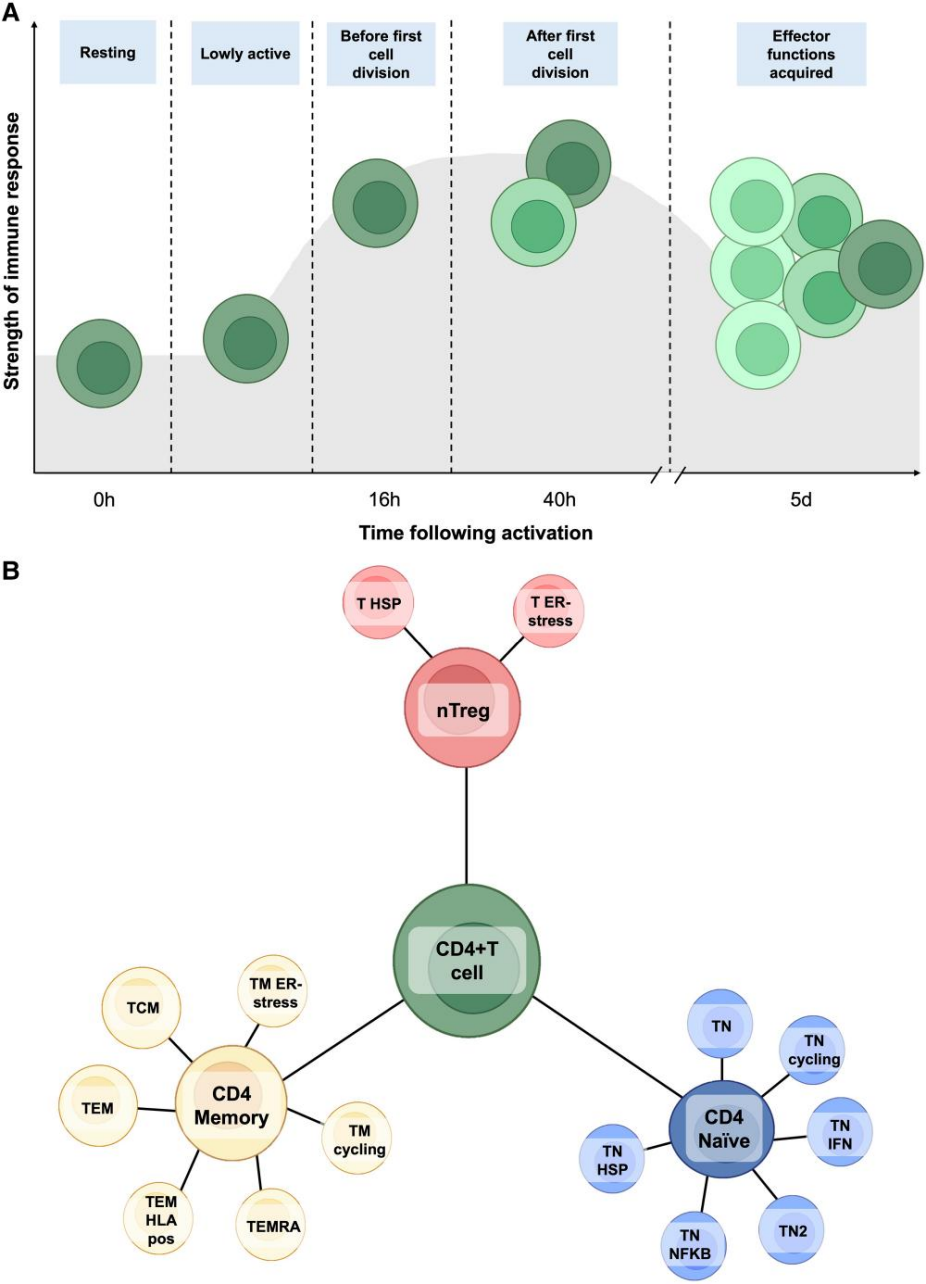
Overview of CD4+ T cell activation states and subtypes A) CD4+ T cells undergo complete remodeling of gene expression to shape their effector function upon activation. This activation occurs in distinct stages, progressing from a resting stage (0 h), to minimally active cells following activation [“lowly active” (LA)], before undergoing cell division (16 h post-activation), after completion of the first cell division (40 h post-activation), and after acquiring effector functions (5d post-activation). Each activation timepoint, or state, reflects a unique functional and transcriptional profile crucial for immune surveillance and response. Color shadings reflect transcriptomic changes. B) Circulating CD4+ T cell subtypes can broadly be grouped into 3 functional clusters: (i) naïve T cells (CD4 naïve)—cells that have not yet encountered an antigen; (ii) memory T cells (CD4 memory)—essential for rapid and robust responses to previously encountered antigens; (iii) regulatory T cells (nTreg)—pivotal for maintaining immune homeostasis by suppressing excessive immune responses.

Among lymphocytes, CD4+ T cells are well-known for their role in anti-tumor responses either through direct action and/or by recruitment of other immune cells.^7^ These cells are strong candidates for contributing to the protective effect of lymphocyte count against CRC risk. Upon activation, CD4+ T cells undergo extensive gene expression remodeling to shape their effector function.^8^ This activation process occurs through distinct stages (Fig. 1A), beginning in a resting state (0 h), followed by a minimally active stage post-activation [“lowly active” (LA)], progressing through cell division (16 h post-activation), post-division (40 h post-activation), and culminating in the acquisition of effector functions (5 d post-activation).^8^ Each of these stages is characterized by unique functional and transcriptional profiles critical for immune surveillance and response.

CD4+ T cells are also significantly heterogeneous across their subtypes.^8^ Circulating CD4+ T cell subtypes can be broadly grouped into 3 functional clusters: (i) naïve T cells (CD4 naïve)—cells that have not yet encountered an antigen; (ii) memory T cells (CD4 memory)—essential for rapid and robust responses to previously encountered antigens; (iii) regulatory T cells (nTreg)—pivotal for maintaining immune homeostasis by suppressing excessive immune responses (Fig. 1B).^8^ Not all CD4+ T cells undergo all activation stages given some exist in a terminally differentiated state (refer to Table S1 for more information). Despite their critical and distinct roles, most studies examining CD4+ T cells in CRC have relied on bulk tissue analyses (eg whole blood) which fail to capture gene expression changes of these cells. This limitation obscures the dynamic nature of CD4+ T cells and hinders our ability to identify the biological mechanisms driving the protective effects of lymphocytes on CRC development.

Here, we aimed to identify key immune-related genetic drivers of CRC risk with potential to inform novel prevention or therapeutic strategies. To achieve this, we leveraged summary genetic data capturing associations between germline variants and single-cell transcriptomic data, allowing us to investigate gene expression across dynamic CD4+ T cell subtypes and activation timepoints. We performed transcriptome-wide Mendelian randomization analyses (MR), which, under certain assumptions, can provide causal estimates,^9^ to identify genes that may play a role in CRC development based on single-cell transcriptomic data generated previously from CD4+ T cells.^8^ We then performed genetic colocalisation to evaluate possible misinference due to linkage disequilibrium and add robustness to our results. Additionally, we applied an additional sensitivity analysis to determine whether the genetic instruments used to proxy gene expression in CD4+ T cells in MR analyses are also associated with gene expression in other tissues, such as colorectal tissue. Our approach combining causal methodologies with single-cell transcriptomic data not only advances our understanding of the protective roles of CD4+ T cells in CRC risk, but also highlights potential therapeutic targets for CRC prevention.

## 2. Methods

**Fig. 2.**
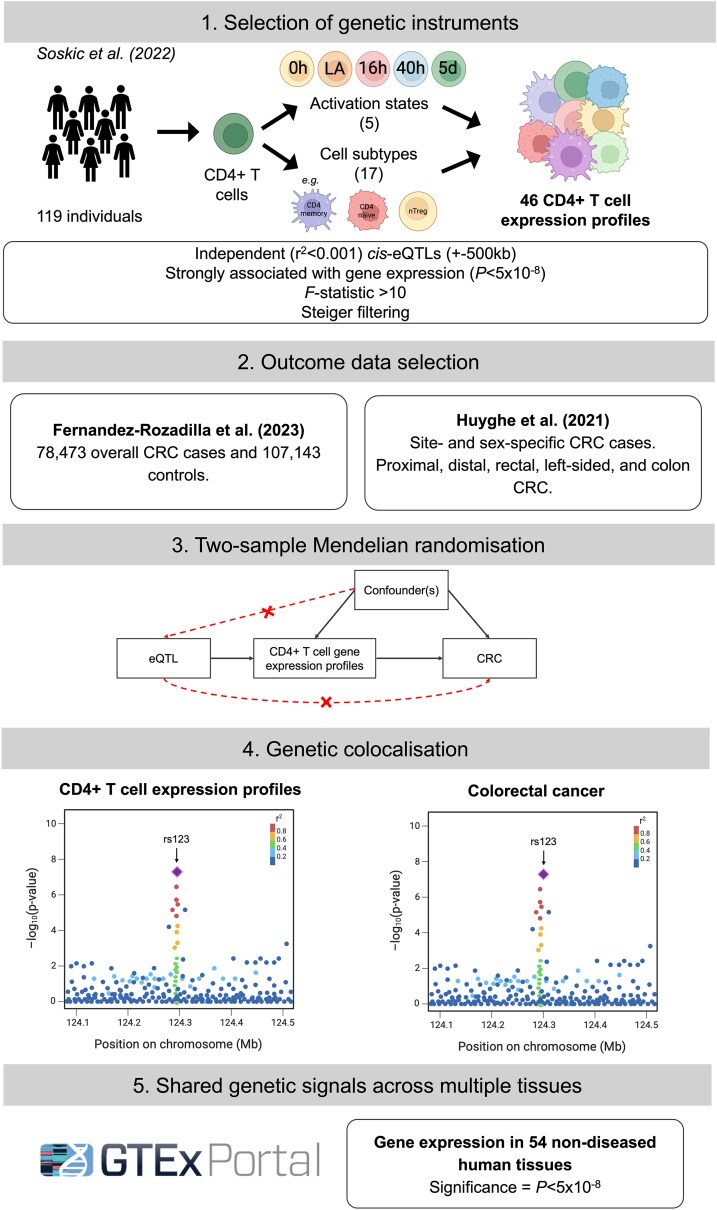
Flow chart describing study methodology. Note that genetic instruments were not available for all cell subtypes at every activation timepoint, meaning the total number of CD4+ T cell expression profiles investigated does not exactly equal the number of timepoints^5^ multiplied by the number of cell subtypes.^17^ CRC, colorectal cancer; eQTL, expression quantitative trait loci; GTEx, Genotype-Tissue Expression.

To identify key gene expression alterations across subtypes of CD4+ T cells with a role in CRC development, we conducted summary-level MR analyses. To evaluate the robustness of our results to misinference by linkage disequilibrium (LD), we then performed genetic colocalisation. For genes with robust evidence for an effect on CRC risk across both analyses, we performed a sensitivity analysis to evaluate the possibility of bias arising from horizontal pleiotropy through gene expression changes in other tissues. An overview of our methodology is represented in Fig. 2.

### Ethics statement

All GWAS obtained approval from the appropriate ethical committee(s).^8,10–12^

## 3. Study populations

### 3.1 CD4+ T cell gene expression genome-wide association study (GWAS)

Soskic et al.^8^ isolated peripheral mononuclear cells (PMBCs) from blood samples obtained from 119 healthy individuals (56% male; 44% female) of European (“British”) ancestries (mean age 47 ± 15.61). CD4+ T cells were then further isolated from the PBMCs and activated with anti-CD3/anti-CD28 human T-Activator Dynabeads (Invitrogen) at a 1:2 beads-to-cells ratio. This resulted in a total of 655,349 CD4+ T cells across all individuals available for the single-cell transcriptomics.^8^

We used summary-level expression quantitative trait loci (eQTL) results derived from single-cell transcriptomic data.^8^ These eQTLs were identified from the transcriptomes of CD4+ T cells sampled at 5 distinct timepoints following the *ex vivo* activation described above, representing a spectrum from resting to activated states (Fig. 1A). The timepoints included resting cells (0 h), lowly active (LA), before undergoing cell division (16 h post-activation), after the first cell division (40 h post-activation), and after acquiring effector functions (5 d post-activation).^8^ Additionally, unsupervised clustering was performed by Soskic et al.^8^ using the transcriptomic data to group cells based on gene expression patterns and activation markers across the activation timepoints. This process identified 17 distinct CD4+ T cell subtypes which fall into 3 main groups—CD4 memory, CD4 naïve and nTreg (Fig. 1B). Combining the 17 cell types with different activation timepoints, Soskic et al.^8^ described a total of 46 distinct CD4+ T cell gene expression profiles (eg CD4_naive_0h represents CD4 naïve cells immediately following activation; note that all 5 activation timepoints are not available for every subtype, as they can become terminally activated before reaching the final timepoint). All CD4+ T cell subtypes included in our analyses are described in Table S1.

### 3.2 CRC risk GWAS

We used summary-level data obtained from the largest available GWAS of CRC risk in European ancestries^10^ (N cases = 78,473; *N* controls = 107,143) and the largest available GWAS of sex-specific and site-specific CRC risk in European ancestries^11,12^: proximal (14,416 cases, 43,099 controls), distal (12,879 cases, 43,099 controls), rectal (14,150 cases, 43,099 controls), left-sided (27,004 cases, 43,099 controls), colon (28,736 cases, 43,099 controls), female (24,594 cases, 23,936 controls), and male (28,271 cases, 22,351 controls). CRC classification was determined using ICD-10 codes, with most cases being newly diagnosed. CRC subsites were categorized based on location: colon cancer includes the proximal colon (any primary tumor arising in the cecum, ascending colon, hepatic flexure, or transverse colon), the distal colon (any primary tumor arising in the splenic flexure, descending colon, or sigmoid colon), and cases with an unspecified site. Rectal cancer includes any primary tumor arising in the rectum or rectosigmoid junction.

### 3.3 MR analyses

MR uses genetic variants, typically single nucleotide polymorphisms (SNPs), which under specific assumptions can be used in an instrumental variable framework to obtain causal estimates. The 3 core assumptions are: (i) the genetic variant(s) must be associated with the exposure; (ii) there are no confounders of the association between the genetic variant(s) and the outcome; and (iii) the genetic variant(s) is/are only associated with the outcome via an association with the exposure.^9^ In addition to these core assumptions, additional assumptions, such as exposure and outcome data being obtained from nonoverlapping populations from the same underlying population in summary-level MR, also exist^13^; see Sanderson et al.^14^ for a detailed overview of MR assumptions.

We identified cis-eQTLs for each gene as SNPs within the gene coding region (±500 kb) which had a *P* < 5 × 10^−8^ and were independent of other associated SNPs within a 10 kb window using a LD *r*^2^ < 0.001. We excluded weak instruments using an *F*-statistic < 10^15^ and performed Steiger filtering^16^ to exclude SNPs which may explain more variance in the exposure than the outcome, to avoid bias from potential reverse causation. As such, we identified 10,994 cis-SNPs associated with expression of 1,805 genes across the 46 CD4+ T cell gene expression profiles. For all MR analyses we used the Wald ratio to obtain causal estimates and the delta method to approximate standard errors.^17^ Benjamini-Hochberg correction (<0.05) was applied as a false discovery rate (FDR)-correction.^18^

This manuscript was written following the STROBE-MR guidelines.^19,20^ A completed STROBE-MR checklist is included in the Supplementary Information.

### 3.4 Genetic colocalisation analyses

Genetic colocalisation uses GWAS summary statistics to distinguish between distinct causal variants underlying a shared causal signal at a specific locus for 2 (or more) traits.^21^ Genetic colocalisation evaluates the posterior probability of 5 mutually exclusive scenarios: H0: there are no variants in the given genomic region causal to either trait; H1: there is a causal variant in the given genomic region for the first but not second trait; H2: there is a causal variant in the given genomic region for the second but not the first trait; H3: there is a causal variant in the given genomic region for both traits, but this variant is different between the traits; and H4: the causal variant in the given genomic region is the same for both traits.^21,22^

To evaluate the possibility of misinference due to LD in our MR analyses, we performed pair-wise conditional colocalisation (PWCoCo) for all genes meeting our predetermined threshold (FDR-*P* < 0.05) in MR analyses. Briefly, PWCoCo performs conditional and joint multi-SNP analysis (GCTA-COJO) to detect independent associations within a region. To achieve this, PWCoCo conditions each SNP on the sentinel SNP to identify conditionally independent SNPs for which colocalisation is then performed.^23^ Thus, in contrast with other genetic colocalisation methods, this approach retains the single causal variant assumption but allows for the testing of multiple causal variants within a genomic region. By combining our MR analyses with PWCoCo, we were therefore able to evaluate whether MR evidence was likely being driven by LD between distinct causal SNPs for gene expression and CRC risk; a possible violation of the third core MR assumption. Colocalisation was performed using PWCoCo for all SNPs within ±500 kb of the gene coding region using prior probabilities (*p*1 = *p*2 = 1 × 10^−5^ and *p*12 = 1 × 10^−7^) based on ∼1,621 SNPs present within each window (suitable prior probabilities chosen on an online calculator: https://chr1swallace.shinyapps.io/coloc-priors/, shown in Fig. S1). We interpreted posterior probabilities as a scale of evidence for a shared causal variant and set a threshold of H4 > 0.8 as supporting evidence for colocalisation.

### 3.5 Shared genetic signals across multiple tissues

We evaluated whether evidence for a causal effect of gene expression on CRC risk from our MR and colocalisation analyses were specific to CD4+ T cells or whether the eQTLs used as genetic proxies may also instrument gene expression in other tissues. Where genetic instruments are associated with expression of the relevant gene in multiple tissues, this could suggest possible bias in our MR results from horizontal pleiotropy, a violation of the exclusion restriction assumption (ie the pathway from genetic instrument to CRC risk would not be through the presumed exposure of gene expression in CD4+ T cells specifically). This would be particularly pertinent if an eQTL was also associated with expression of the gene in the colon tissue itself, given the likely importance of local gene expression in disease development. To investigate this, we obtained summary statistics from the Genotype-Tissue Expression Program (GTEx)^24^ for tissue-specific gene expression for all prioritized genes (ie those with FDR-*P* < 0.05 in MR analyses and H4 > 0.8 in genetic colocalisation analyses). GTEx is a comprehensive public resource that maps how genetic variation influences gene expression across 54 nondiseased human tissues, based on samples from nearly 1,000 cadavers (sample size varies by tissue; see^24^ for more information). We evaluated whether there was evidence for an association between the genetic instruments and expression of the instrumented gene in any of the available tissues in GTEx. We defined evidence of an association as genome-wide significance (*P* < 5 × 10^−8^). Where the genetic instrument used in MR analyses was not available in the GTEx dataset, we instead evaluated the SNP in highest LD with the eQTL which was available in GTEx data (minimum required *r*^2^ = 0.8).

## 4. Results

### 4.1 MR analyses

**Fig. 3.**
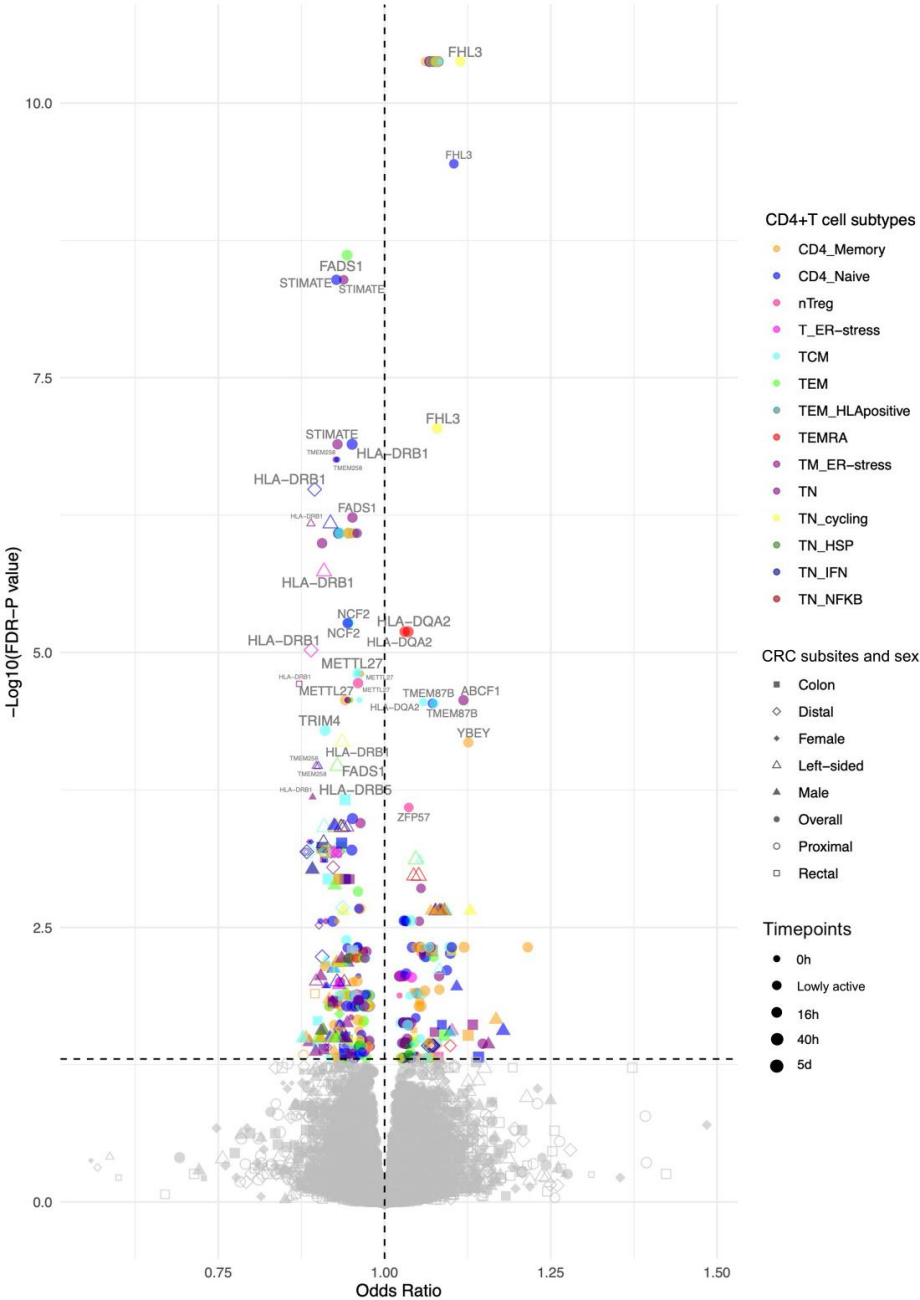
Volcano plot showing 2-sample MR results. Colors represent the different CD4+ T cell subtypes, shapes represent CRC subsites and sex, and point size represents activation state. Points labeled with gene names. Refer to Table S1 for CD4+ T cell subtype definitions and abbreviations.

**Fig. 4.**
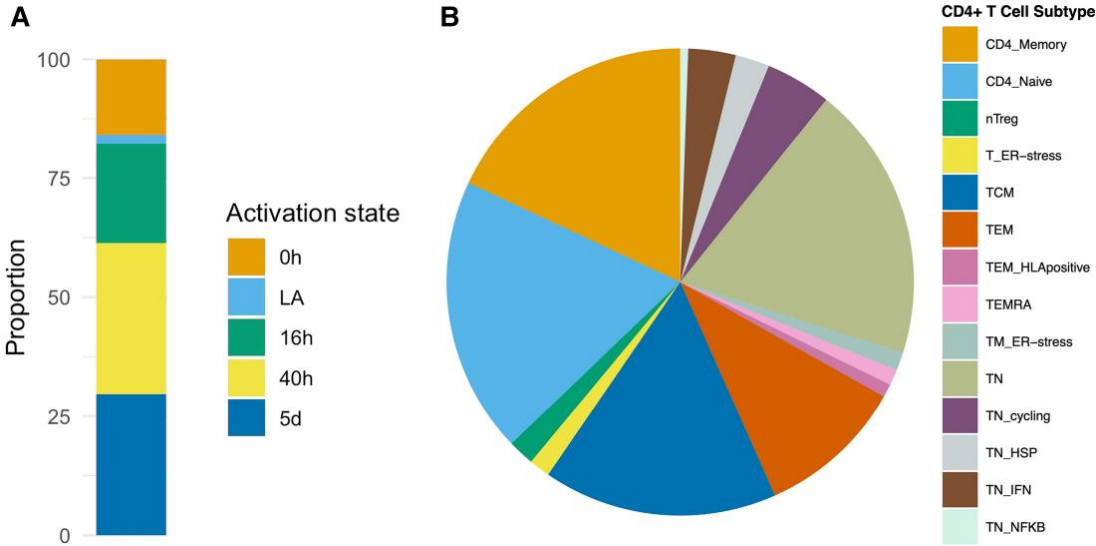
Proportion of CD4+ T cell activation states and cell subtypes contributing to significant MR results. A) Stacked bar plot representing proportion of CD4+ T cell activation states (0 h, LA, 16 h, 40 h, 5 d) contributing to main study. B) Pie chart representing proportion of CD4+ T cell subtypes contributing to main study results. Refer to Table S1 for CD4+ T cell subtype definitions and abbreviations. LA, lowly active.

Results are given as odds ratio (OR) of CRC risk per standard deviation (SD) higher expression of the gene in CD4+ T cells. Of 1,805 genes (across the 46 CD4+ T cell gene expression profiles), expression of 61 genes had evidence (FDR-*P* < 0.05) for a potential causal effect on CRC risk (Fig. 3; Table S2). Among these, the activation state and cellular subtype that contributed the most were CD4+ T cells 40 h post-activation (Fig. 4A) and CD4 naïve respectively (Fig. 4B).

### 4.2 Genetic colocalisation analyses

Of the 61 genes with evidence for an association between their expression and CRC risk, 6 had evidence for a shared signal with CRC risk (H4 > 0.8): *FADS2, FHL3, HLA-DRB1, HLA-DRB5, RPL28, and TMEM258* (Table 1; full results in Table S4).

**Table 1.** Genetic colocalisation results.

	Gene					
	*FADS2*	*FHL3*	*HLA-DRB1*	*HLA-DRB5*	*RPL28*	*TMEM258*
Overall		CD4 Memory (0 h, 16 h, 40 h, 5d); TN cycling (40 h); CD4 Naive (16 h, 40 h, 5 d); TCM (0 h, 16 h, 5 d); TN (16 h, 40 h, 5 d); TN HSP (5 d); TEM (40 h)	TM ER-stress (40h); CD4 Naive (5d); TEM (0h)		TCM (0 h); CD4 Naive (5 d)	
Sex						
Male	TN IFN (5d)	TCM (0 h); CD4 Memory (0 h, 16 h); TN HSP (5 d); CD4 Naive (40 h); TN (40 h); TEM (40 h)	TM ER-stress (40 h)			
Female						CD4 Naive (0 h); TN (0 h)
CRC subsites						
Colon				TCM (5 d)		
Distal			TM ER-stress (40 h)			
Left-sided			TM ER-stress (40 h)			
Proximal						
Rectal						

Results of genetic colocalisation analysis using PWCoCo. CRC, colorectal cancer; PWCoCo, PairWise Conditional and Colocalisation; refer to Table S1 for CD4+ T cell subtype definitions and abbreviations.

### 4.3 Shared genetic signals across multiple tissues

For the 6 genes with robust evidence from MR (FDR-*P* < 0.05) and genetic colocalisation (H4 > 0.8) analyses, we evaluated whether these observations could be explained through gene expression in tissues other than CD4+ T cells. Table 2 summarizes the results from this sensitivity analysis.

**Table 2.** Shared genetic signals across multiple tissues.

Gene	SNP		Genome-wide significant threshold^a^ reached		
	Lead	LD (*R*^2^)	Sigmoid colon tissue	Transverse colon tissue	All tissues (%)
*FADS2*	rs61897793	–	Y	Y	61.7
*FHL3*	rs67631072	–	N	N	73.0
*HLA-DRB1*	rs3104393	rs9272025 (0.993)	Y	Y	69.6
*HLA-DRB5*	*NA*	*NA*	*NA*	*NA*	*NA*
*RPL28*	rs4806665	–	N	Y	61.1
*TMEM258*	rs174538	–	Y	N	52.0

Associations of eQTLs and gene expression in colon tissue with a comparison to all other tissues using GTEx data. GTEx, Genotype-Tissue Expression; LD, linkage disequilibrium; SNP, single nucleotide polymorphism.

^a^Significance defined as genome-wide threshold = <5 × 10^−8^; *NA* indicates that both the lead SNP and a SNP in high LD was not available in the GTEx dataset with a suitable proxy.

The lead SNP and any SNPs in high LD for *HLA-DRB5* expression were not available in GTEx, meaning we were unable to include this gene in this sensitivity analysis. Overall, the lead SNPs for all 5 other genes with robust evidence for a role in CRC risk were associated with gene expression in other tissues in the GTEx dataset (*P* < 5 × 10^−8^). The proportion of tissues in which eQTLs were associated with gene expression ranged from 52% to 73% (mean = 64%), making it difficult to decipher the relevant tissue through which the identified genes act to influence CRC risk. For 3 of these genes (*FADS2, HLA-DRB1, RPL28*), the tissues where the eQTL associated with gene expression included transverse and sigmoid colon, highlighting a biologically plausible mechanism of horizontal pleiotropy in our analyses.

## 5. Discussion

We used a causal framework employing MR and genetic colocalisation to investigate whether CD4+ T cell subtype- and activation timepoint-specific gene expression may have a causal role in CRC risk. We identified 6 genes (*FADS2, FHL3, HLA-DRB1, HLA-DRB5, RPL28,* and *TMEM258*) with robust evidence for a causal role. Notably, *TMEM258,* not been previously implicated to CRC, emerged as a novel candidate. Several findings were specific to different CD4+ T cell activation states (eg prior to antigen presentation). However, the lead genetic variants associated with these 6 genes in CD4+ T cell subtypes at different activation points were also associated with expression in multiple other tissues, for some genes including colon tissues, suggesting the effect of gene expression on CRC risk might not be entirely specific to CD4+ T cells.

**Fig. 5.**
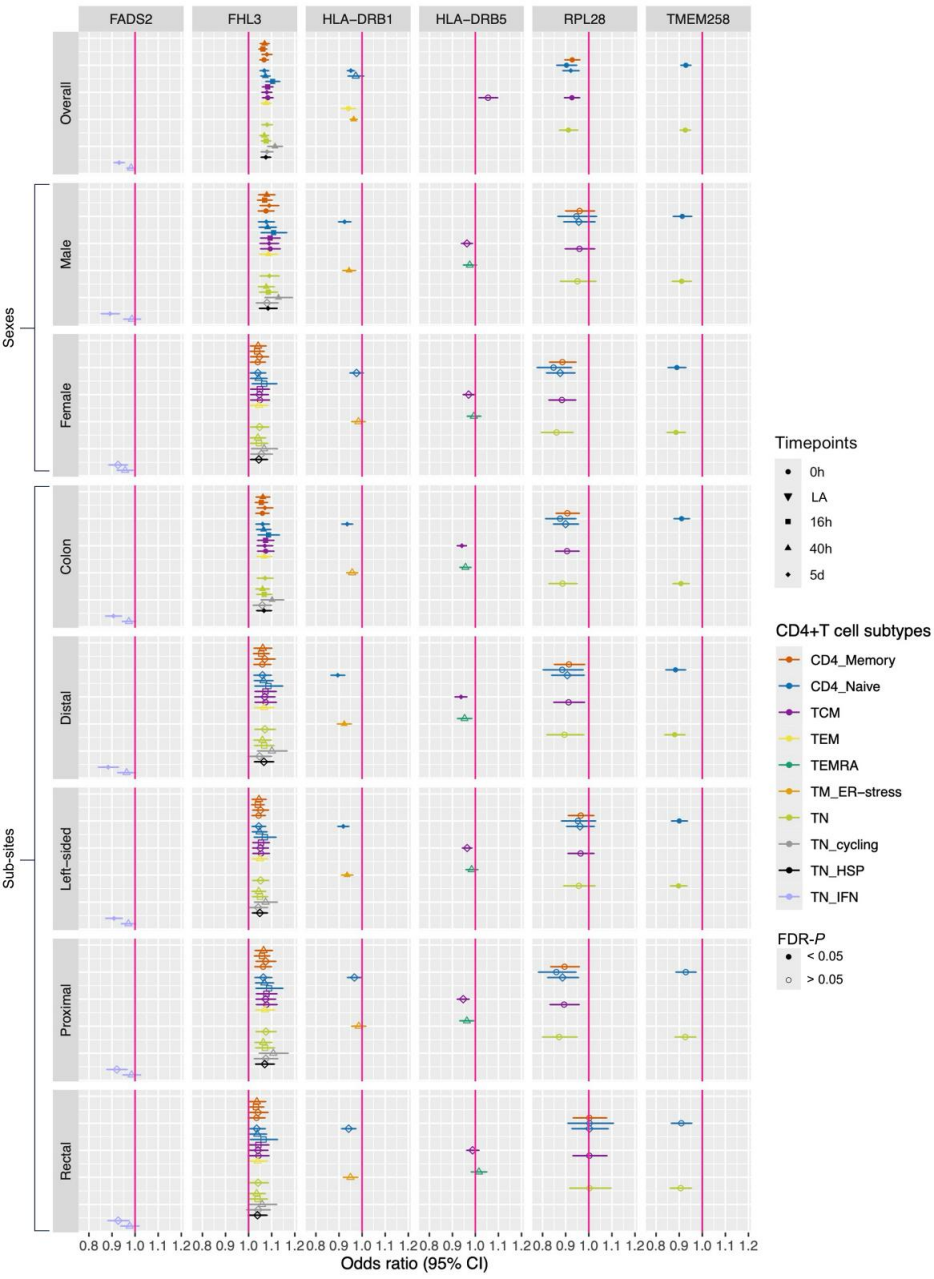
MR results for genes that showed strong evidence of genetic colocalisation, separated by gene, sex and CRC subsites. Colors represent CD4+ T cell subtypes; shapes represent activation timepoint; and point fill represents whether FDR-*P* is < or > 0.05. CRC, colorectal cancer; LA, lowly active.

We observed evidence for sex-specific effects of gene expression in our analyses (Fig. 5; Table S3). For example, MR results suggested a protective effect of higher *TMEM258* expression on female-specific CRC risk in CD4 naïve cells (OR = 0.89, confidence interval (CI) = 0.85–0.93) and TN cells at rest (OR = 0.89, CI = 0.85–0.93) but not on male-specific CRC risk. *TMEM258* is involved in protein synthesis, folding and trafficking.^25^ Previous research has demonstrated that dysregulation of *TMEM258* expression can lead to endoplasmic reticulum (ER) stress, consequently triggering activation of the unfolded protein response (UPR).^25^ UPR is known to be beneficial to CD4+ T cells as it supports differentiation, activation, cytokine production and autophagy.^26^ This may explain the potential mechanism by which increased *TMEM258* expression could reduce CRC risk. Additionally, we observed sex-specific effects for *FADS2,* as its expression was significantly protective against male-specific CRC development. *FADS2*, which encodes the enzyme fatty acid desaturase 2, plays a crucial role in the biosynthesis of polyunsaturated fatty acids (PUFAs), including omega-3 and omega-6 fatty acids.^27^ While omega-3 PUFAs have been shown to have protective associations against various cancers, including CRC, the relationship between omega-6 PUFAs and CRC risk remains unclear.^28–31^ We found evidence for a protective effect of *FADS2* expression on male CRC risk in naïve T cells producing interferon-gamma (IFN) upon activation (TN IFN) 5 days post-activation (OR = 0.89, CI = 0.85–0.93). Metabolic reconstruction, mediated by gene expression changes, is an important aspect of CD4+ T cell activity.^32,33^ Moreover, we observed null results for an effect estimate of *FADS2* expression in the same cell type 40 h post-activation (Fig. 5), which supports an activation timepoint-specific effect of this gene and demonstrates the importance of considering the dynamic nature of CD4+ T cells.

In addition, we found evidence for a protective effect of 2 genes involved in the human leukocyte antigen (HLA) complex, *HLA-DRB1 and HLA-DRB5*, in CRC risk. We found evidence for a causal effect of higher *HLA-DRB1* expression on overall, male, distal and left-sided CRC risk in several subtypes of CD4+ T cells, particularly memory T cells experiencing ER stress (TM ER-stress) at 40 h post-activation (Fig. 5). Cancer of the distal colon, which is part of left-sided CRC, is disproportionately diagnosed in males, which reflects these results. HLA-DRB1 is a class II major histocompatibility complex (MHC) protein involved in aiding the immune system distinguishing the body's own proteins from those made by foreign bodies.^34^ The HLA-DRB1 chain is a component of the MHC class II complex on the surface of antigen-presenting cells and is responsible for antigen presentation to CD4 T helper cells, thus supporting immune activation in response to peptides from pathogens.^34^ Our results suggest that the beneficial effect we observe of increased expression of this gene on CRC may be related to enhanced antigen presentation to CD4+ T cells, consequently boosting anti-tumor immunity. We found strong evidence for a protective effect of *HLA-DRB5* expression on colon-specific CRC risk in memory T cells (TCM) 5 d post-activation (OR = 0.94, CI = 0.92–0.96). Similar to *HLA-DRB1, HLA-DRB5* is an MHC class II protein.^34^ However, unlike *HLA-DRB1, HLA-DRB5* is only expressed in a subset of the population.^35^ This protein is involved in presenting peptides to T-helper cells, aiding in the initiation of an immune response.^34^ These findings reinforce the importance of immune vigilance/surveillance and its protective effect against CRC development.^36^ Higher *HLA-DRB5* expression may also enhance antigen presentation, resulting in stronger and more specific anti-tumor responses. Our results (Fig. 5) show that higher *HLA-DRB5* expression in TCM cells 5 d post-activation has a protective effect risk of colon cancer, which aligns with this explanation. While we observed evidence for a protective effect of *HLA-DRB5* expression on colon-specific CRC risk in TCM cells, key players in long-term immune memory and response,^37^ we found little evidence for similar effect in effector memory cells re-expressing CD45RA (TEMRA). This highlights a cell type-specific relationship and underscores the importance of considering distinct subtypes of CD4+ T cells.

We observed a protective effect of higher *RPL28* expression on overall CRC risk in TCM cells at rest (OR = 0.92, CI = 0.89–0.96) and CD4 naïve cells 5 d post-activation (OR = 0.89, CI = 0.82–0.96). The *RPL28* gene encodes for a ribosomal protein component of the large ribosome subunit (60S) and is involved in the assembly of ribosomes and the translation of messenger ribonucleic acid (mRNA) into proteins.^38^ As TCM cells play an important role in long-term immune memory and surveillance, *RPL28* may facilitate the production of necessary proteins that contribute to their maintenance and longevity. Similarly, CD4+ naïve T cells at 5 d post-activation require increased protein synthesis in order to support rapid proliferation and differentiation.^8^

Finally, we identified strong evidence for a detrimental effect of higher *FHL3* expression on both overall and male-specific CRC risk across several subtypes of CD4+ T cells during rest and at 3 post-activation timepoints (Fig. 5). *FHL3* encodes the 4 and a Half LIM Domains 3 (FHL3) protein, which is highly expressed in skeletal muscle. Although its specific function in CD4+ T cells is unclear,^39^ being localized to the nucleus, it is believed to play a critical role in transcription regulation in other cell types.^40^ Differential expression of FHL3 could plausibly influence downstream gene expression involved in T cell function. *FHL3* expression across several immune cell subtypes was found to increase risk of CRC in a recently published MR analysis, and FHL3 expression in CRC tumors was also found to predict overall survival.^41^ However, the carcinogenic role of FHL3 appears complex, with both pro and anti-tumorigenic effects reported across different cancer types.^40,42–44^ FHL3 has been shown to interact with key transcription factors such as cyclic adenosine monophosphate (cAMP) response element binding protein (CREB) and Supressor of Mothers against Decapentaplegic (SMAD) proteins, affecting downstream genes involved in cell proliferation, apoptosis, and immune signaling.^40^ We can speculate that aberrant FHL3 expression in CD4+ T cells could disrupt downstream signaling processes such as cytokine signaling or impair effective anti-tumor immunity, thus promoting a pro-tumor microenvironment, though further functional research is necessary to elucidate specific mechanisms through which FHL3 may confer the detrimental effects suggested by our results.

Our study combined 2 complementary methods which, taken together, provide evidence for a causal relationship ie robust to biases and confounding factors commonly associated with traditional epidemiological studies.^9^ Given the complexity and dynamic nature of CD4+ T cells, we aimed to identify genes with a role in CRC risk using single-cell data spanning a range of cell subtypes and activation points. However, several limitations to our analysis exist. First, we used data from European ancestries which, though we assume are homogenous and therefore satisfy our genetic instrument assumptions, means these results may not be generalizable to other populations. Second, sensitivity analyses revealed that genetic variants associated with gene expression in CD4+ T cells were also linked to gene expression in other tissues, suggesting that our findings may reflect broader tissue-level expression changes rather than being CD4+ T cell-specific. Third, 2 of the identified genes, *HLA-DRB1* and *HLA-DRB5*, are located within the MHC region on chromosome 6, a region known for high genetic variability and LD due to its complex genetic architecture. While biologically relevant, this variability may introduce biases to our study,^45,46^ consequently warranting cautious interpretation on findings related to these genes. Future methodologies may better resolve gene colocalisation within the MHC region.^47^ Fourth, though we investigated our outcome stratified by sex, it was not possible to do this for our exposures and it is unclear whether sex is an important factor in the genetic architecture of gene expression. Fifth, we acknowledge that, according to STROBE-MR guidelines, an ideal approach would include a replication dataset to validate our results, though this was not feasible given the novelty of the underlying data. Lastly, while MR is increasingly accessible via large public GWAS datasets, this ease-of-use has raised concerns surrounding the proliferation of studies with insufficient triangulation or methodological rigor.^48–50^ We have taken steps to minimize such limitations, such as applying MR within single-cell resolution data and adhering to STROBE-MR guidelines, but we recognize that MR alone is insufficient to definitively establish causality.^48–50^ In particular, applying MR to intermediate molecular traits risks violating MR core assumptions, as genetic variants could affect gene expression in multiple cell types or tissues, complicating interpretation of causal estimates.^51^ Therefore, our findings should be viewed as hypothesis-generating and should ideally be validated using other, complementary methodological approaches. Despite these limitations, our work has potential translational value. By identifying the expression of genes in CD4+ T cells that may causally influence CRC risk, we prioritize genes and pathways for functional validation and future possible therapeutic targeting.

## 6. Conclusion

Our analysis identified 6 genes with robust evidence for a causal effect of expression in CD4+ T cell subtypes on CRC risk, including *TMEM258*, a gene not previously reported in relation to CRC development. This highlights its potential as a novel candidate for further research into CRC pathogenesis. Additionally, our findings revealed significant variability in causal estimates of CRC risk across different CD4+ T cell subtypes, activation time points, CRC anatomical subsites, and sex. These observations underscore the complex, context-dependent relationships between immune system dynamics and CRC risk.

## Supplementary Material

qiaf131_Supplementary_Data

## Data Availability

Statistical analyses: The bulk of the analyses were performed using RStudio (version 2024.4.2.764).^52^ MR analyses were performed using TwoSampleMR (version 0.5.7).^53^ Data was manipulated using the following packages: arrow (version 16.1.0), biomaRt (version 2.58.0), data.table (version 1.14.10), dplyr (version 1.1.4), GenomicRanges (version 1.54.1), gwascat (version 2.34.0), gwasvcf (version 0.1.2), rtracklayer (version 1.62.0), stringr (version 1.5.1), tidyr (version 1.3.0).^54–64^ Plots were created using ggrepel (version 0.9.5) and ggplot2 (version 3.4.4).^65,66^ Allele frequencies were calculated using PLINK2.0.^67^ Genetic colocalisation analyses were performed using Cmake (version 3.20.0) and PWCoCo (version 1.0).^23^ Priors used in the colocalisation analyses were computed using https://chr1swallace.shinyapps.io/coloc-priors/.

## Data and code availability

All data can be found in the manuscript, in the Supplementary Information, or in the links provided in the references. The GWAS of overall CRC in European ancestries can be accessed using the GWAS catalogue (https://www.ebi.ac.uk/gwas/) accession no. GCST90129505. The data where the genetic instruments were extracted for all MR analyses are available on this link (https://trynkalab.sanger.ac.uk). All code used to carry out analyses has been made publicly available on GitHub (https://github.com/bennydeslandes/CD4-T_cell_CRC). Further information on the TwoSampleMR package and PWCoCo can be found on https://github.com/MRCIEU/TwoSampleMR/, and https://github.com/jwr-git/pwcoco, respectively.
